# Serum Biomarkers for Nutritional Monitoring in Motor Neuron Disease: A Longitudinal Cohort Study

**DOI:** 10.3390/nu18172913

**Published:** 2026-09-04

**Authors:** Sarah A. Roscoe, Scott P. Allen, Christopher J. McDermott, Theocharis Stavroulakis

**Affiliations:** Division of Neuroscience, School of Medicine and Population Health, Sheffield Institute for Translational Neuroscience, University of Sheffield, Sheffield S10 2HQ, UK; s.roscoe@sheffield.ac.uk (S.A.R.); s.p.allen@sheffield.ac.uk (S.P.A.); c.j.mcdermott@sheffield.ac.uk (C.J.M.)

**Keywords:** nutritional biomarkers, serum, motor neuron disease, amyotrophic lateral sclerosis, nutritional assessment

## Abstract

**Background:** Malnutrition and metabolic dysregulation are common in motor neuron disease (MND) and contribute to accelerated functional decline and reduced survival. Routine serum biochemical analytes can be used to assess nutritional status; however, their interpretation in MND is confounded by systemic inflammation and disease-related metabolic changes. This study investigated the relationship between serum biochemical analytes, inflammatory status, disease severity, progression, and body composition in people living with MND. **Methods:** In this single-centre, longitudinal prospective cohort study, 19 participants with confirmed MND were assessed at enrolment and at three-month intervals up to nine months. Serum concentrations of albumin, prealbumin, creatinine, transferrin, ferritin, retinol-binding protein, and lipid fractions were measured alongside routine inflammatory markers. Disease severity and progression were assessed using the revised ALS functional rating scale and King’s College Staging System. Anthropometry included percentage weight change, body mass index, mid-upper arm circumference, triceps skinfold thickness, arm muscle area, and calf circumference. Participants with evidence of baseline inflammation were excluded from nutritional analyses. **Results:** Low-grade systemic inflammation was present in 9/19 (47%) of participants at enrolment, most commonly reflected by elevated fibrinogen. In the non-inflammatory sub-cohort (*n*/N = 10/19), serum creatinine was positively correlated with muscle-related functional subscores (*p* = 0.007) and declined between enrolment and three months (*p* = 0.02), showing predominantly negative trajectories by nine months. HDL cholesterol also declined over the nine-month follow-up (*p* = 0.03) and lipid fractions correlated positively with disease stage (*p* <0.001–0.02), suggesting evolving metabolic stress. Elevated serum retinol-binding protein was observed in 90% (*n*/N = 9/10) of participants without inflammation. Biochemical evidence of malnutrition (low transferrin and/or creatinine) was detected in one-third of participants, accompanied by weight loss and reductions in limb anthropometric parameters. Malnutrition risk was identified in 1/10 (10%) of the non-inflammatory sub-cohort using a modified ESPEN-based framework, and in 2/10 (20%) participants using GLIM criteria. **Conclusions:** Routine serum biochemical analytes provide complementary information on nutritional and metabolic status in MND when interpreted alongside inflammatory markers and anthropometry. Serum creatinine emerged as a longitudinal marker of muscle wasting, reflective of denervation and nutritional depletion, while lipid and retinol-binding protein alterations highlight non-nutritional disease mechanisms that may confound standard biomarker interpretation, informing more targeted nutritional assessments. These findings support a multimodal approach to nutritional monitoring in MND and emphasise the importance of accounting for inflammatory status in biomarker interpretation. Given the small sample size and the limited representation of non-ALS phenotypes, these findings should be regarded as hypothesis-generating and require confirmation in larger, multicentre cohorts with balanced phenotypic representation.

## 1. Introduction

### 1.1. Malnutrition in Motor Neuron Disease

Motor neuron disease (MND) is an incurable heterogeneous group of progressive neurodegenerative conditions marked by the gradual degeneration and eventual loss of motor neurons [1]. The resulting weakness and wasting affects muscles controlling movement, speech and breathing, and most patients die of respiratory failure within approximately two-to-three years of diagnosis [2,3]. The rate of disease progression depends on age, site of onset and MND phenotype [2].

Malnutrition and associated weight loss are common in MND, estimated to affect 16–55% of people living with MND [4,5]. Factors such as dysphagia, mastication weakness, and a decreased upper limb mobility and dexterity contribute to a sub-optimal caloric intake [6,7]. At the same time, energy needs are frequently elevated: resting energy expenditure (REE) can exceed the value predicted for a person’s age, weight and sex, a state termed ‘hypermetabolism’ [8,9]. This is estimated to affect 50–68% of people living with sporadic MND [10]. Because hypermetabolism accelerates the breakdown of carbohydrate, lipid and protein stores [11], it compounds the nutritional difficulties already faced by people with MND [12]. Those with the largest energy deficits tend to decline more rapidly and survive for a shorter time [10].

### 1.2. Assessment of Malnutrition

Due to the array of nutritional indices, assessment tools and thresholds utilised to assess malnutrition, the prevalence is difficult to determine in MND [13]. Most cohorts define malnutrition risk in MND using a percentage weight loss of ≥5–10% from premorbid body weight before the onset of MND symptoms [14,15], in addition to body mass index (BMI) thresholds of <18.5 kg/m^2^ [4] or <20 kg/m^2^ [16]. This allows for changes in body composition, experienced in highly catabolic diseases (such as MND), to be detected when patients may have lost up to 10% of their body weight while remaining within a ‘normal’ BMI range. A mid-upper arm circumference (MUAC) of <23.5 cm for both men and women are typically indicative of a BMI < 20 kg/m^2^, and has been used to indicate a risk of malnutrition [17]. A calf circumference below 31 cm—the threshold set by a World Health Organisation expert committee [18]—is an established clinical indicator of sarcopenia and a predictor of physical function and survival in older adults [19]. Malnutrition has also been indicated by observing changes in anthropometric measurements and related indices in MND (e.g., limb skinfold thickness and circumference measurements) [20]; however, this approach is limited, and results must be interpreted with consideration for disease-specific changes in body composition. Serum analyte concentrations below the clinically acceptable reference ranges have previously been used to indicate malnutrition in MND [21].

Anthropometric indices such as MUAC and calf circumference are, however, indirect surrogates of muscle mass and do not permit the direct quantification of low muscle mass or sarcopenia. More specific techniques—including muscle ultrasonography, bioelectrical impedance analysis (BIA), and dual-energy X-ray absorptiometry (DXA)—allow for more precise, quantitative assessment of muscle mass and quality, and are increasingly being applied in MND. Muscle ultrasonography has been used to assess quadriceps thickness and echogenicity in people with ALS, with reduced thickness and greater echo-intensity associated with lower fat-free mass and appendicular skeletal muscle indices, and independently predicting hospital admission risk [22]. Similarly, BIA-derived phase angle and bioelectrical impedance vector analysis have demonstrated reduced cellular integrity and muscle mass in people with ALS relative to healthy controls, supporting their use as sensitive indicators of nutritional and muscle status beyond BMI alone [23]. DXA has also been applied in ALS to directly quantify appendicular lean and fat mass, with loss of fat mass—alongside a generalised decline in lean mass—correlating with faster functional decline, highlighting its potential as a biomarker of disease progression [24]. Despite their greater precision, these techniques require specialist equipment and trained personnel, and can be difficult to implement in people with MND affected by immobility, positioning difficulties, or the need for domiciliary assessment; anthropometric and biochemical proxies therefore remain more widely used in MND research, including the present study, despite their comparatively lower sensitivity for detecting early or subclinical muscle loss.

### 1.3. Biochemical Analytes as Markers of Nutritional Status

Biochemical analytes drawn from whole blood, plasma, serum, or peripheral blood mononuclear cells (PBMCs) sit at the interface of nutrition and metabolism, capturing not only dietary intake but also how macro- and micro-nutrients are absorbed and processed [25,26]. However, these ‘biomarkers of nutritional status’ can be heavily influenced by systemic inflammation, whereby the serum analyte concentration may decline as a result of hepatic reprioritisation during the acute-phase response [27].

Peripheral blood biomarkers have been used to characterise metabolic, inflammatory, and nutritional status in MND, with studies reporting associations between routine biochemical markers—including albumin, prealbumin, transferrin, ferritin, creatinine, and lipid fractions (total cholesterol, HDL cholesterol and LDL cholesterol, and triglycerides)—and measures of disease severity, nutritional decline, and survival [28,29]. These serum analytes have been predominantly investigated for their role as prognostic indicators, rather than for nutritional monitoring [30].

However, a small number of studies have focused purely on the role of biochemical analytes in assessing nutritional status in MND through comparisons against BMI and body composition [31]. The literature remains heterogeneous and at times contradictory, likely reflecting differences in cohort characteristics and study design, and, importantly, not all studies have accounted for inflammatory status when interpreting these biochemical markers, necessitating the cautious interpretation of reported associations [32]. Furthermore, findings relating to systemic inflammation itself remain inconsistent in MND, with some cohorts demonstrating elevated C-reactive protein (CRP), erythrocyte sedimentation rate (ESR), or fibrinogen [33], while others report limited activation of routine inflammatory pathways in MND populations [34,35].

### 1.4. Research Gap

Despite growing recognition for the importance of assessing nutritional status in MND, the evidence supporting the use of serum biochemical analytes for this purpose remains limited. Existing studies rely mostly on the cross-sectional analysis of serum analytes even in cohorts followed longitudinally for clinical outcomes. This approach limits the inference of serum analytes such as albumin, prealbumin, transferrin and creatinine, despite the dynamic and progressive nature of the disease. Interpretation of these analytes is further complicated by MND pathophysiology, including inflammation, hypermetabolism and muscle denervation, all of which may influence circulating concentrations, independent of the effect of nutritional decline. Consequently, it remains unclear whether observed biochemical abnormalities reflect true nutritional deterioration or non-nutritional disease mechanisms; this is a key challenge in this field. The absence of longitudinal studies pairing repeated biochemical measurements alongside body composition assessment represents a critical gap that limits the development of validated, clinically meaningful biomarkers of nutritional status in MND.

### 1.5. Aim

This longitudinal study aimed to evaluate the utility of serial serum biochemical analytes for monitoring trajectories of nutritional status in people living with MND in relation to disease severity, progression, and body composition.

## 2. Materials and Methods

### 2.1. Study Design

This was a single-site, longitudinal, prospective cohort study of patients with a confirmed diagnosis of MND. Participants were recruited from the Sheffield MND Care and Research Centre between October 2021 and August 2022. Clinical and biochemical assessments were undertaken at three-monthly intervals for a maximum of four consecutive time points: Month 0 (M0, enrolment), M3, M6 and M9. Exclusion criteria were limited to the presence of an underlying, unmanaged co-morbidity that would affect survival or metabolic state (independent of MND), or a decision-making incapacity preventing informed consent. Favourable ethical opinion was obtained from the London-Fulham NHS Research Ethics Committee (21/PR/0092).

### 2.2. Assessment of Disease Severity and Progression

Disease severity was assessed using the self-administered revised ALS functional rating scale (ALSFRS-R) [36], mapped to the King’s College staging system (KCSS) [37]. The rate of disease progression (ΔALSFRS-R) was calculated by: (48—ALSFRS-R total score at time of assessment)/disease duration from symptoms onset (months). Disease duration was defined as the interval between participant-reported date of first MND symptom onset and the first study visit (M0), in months. Participants were categorised by the rate of progression (slow: <1.1 point/month; fast: ≥1.1 point/month) [38].

### 2.3. Biosample Collection, Processing and Analysis

Venous blood (up to 75 mL) was drawn aseptically into ethylenediaminetetraacetic acid (EDTA)- and serum-separator tube (SST)-treated vacutainers. Blood was processed at the Medical Laboratory, Northern General Hospital (NGH), Sheffield Teaching Hospitals NHS Foundation Trust. Processed serum was analysed for the following analytes: albumin, prealbumin, creatinine, retinol-binding protein, transferrin, ferritin, lipid profile (total cholesterol, HDL cholesterol, LDL cholesterol, triglycerides) and estimated glomerular filtration rate (eGFR). The concentration of routine inflammatory parameters, including ESR, white cell count, platelets, lymphocytes, fibrinogen and CRP, were also measured to screen for a pro-inflammatory state.

### 2.4. Assessment of Body Composition

All assessments of body composition were conducted by the same researcher. Body weight to the nearest 0.1 kg (SECA 875, SECA Instruments, Ltd, Hamburg, Germany) and height to the nearest 0.1 cm (SECA 213, SECA Instruments, Ltd, Hamburg, Germany) were recorded in light clothing and shoes in an unaided standing position. Participant-reported weight and height measurements were recorded from those unable to stand unaided. Percentage weight change was calculated at each study time point relative to ‘premorbid body weight’, i.e., weight before the onset of MND symptoms: [initial weight (kg) − current weight (kg)/initial weight (kg)] × 100. BMI (kg/m^2^) was calculated using weight (kg)/height^2^ (m), and categorised according to the WHO classification for adults [39].

A non-elasticated anthropometric tape (SECA 201, SECA Instruments, Ltd, Hamburg, Germany) was used to record MUAC to the nearest 0.1 cm [40,41], and a Harpenden skinfold calliper recorded TSF to the nearest 0.2 mm in triplicate [41]; both were taken bilaterally at the mid-point between the acromion and olecranon processes. AMA (cm^2^) was calculated by: [MUAC (cm) − (TSF (cm) × π)]^2^/(4 × π) [42]. Calf circumference was likewise recorded bilaterally, to the nearest 0.1 cm, at the point of greatest circumference with the participant seated and the knee flexed at 90° (SECA 201). Due to the potential asymmetrical muscle-wasting pattern in MND, a reduction in either contralateral limb measurement was considered as evidence of malnutrition risk.

### 2.5. Criteria for Identifying Participants at Risk of Malnutrition

In the absence of a validated, MND-specific criteria for identifying malnutrition in MND, the Global Leadership Initiative on Malnutrition (GLIM) criteria [43] was used as a comparator against a modified version of the diagnostic framework proposed by Cederholm et al. in the 2015 European Society for Clinical Nutrition and Metabolism (ESPEN) consensus statement [44].

#### 2.5.1. Global Leadership Initiative on Malnutrition (GLIM) Criteria

Identification of malnutrition utilising the GLIM criteria requires at least one phenotypic criterion and at least one etiologic criterion to be met [43]. Phenotypic criteria were: (1) low body mass index (BMI), using age-stratified thresholds (<20 kg/m^2^ [moderate] or <18.5 kg/m^2^ [severe] for age <70 years; <22 kg/m^2^ [moderate] or <20 kg/m^2^ [severe] for age ≥70 years); (2) weight loss, calculated relative to premorbid body weight (10–20% [moderate] or >20% [severe] over ≥ six-months); and (3) reduced muscle mass, assessed using mid-upper arm circumference (MUAC; <23.5 cm) and calf circumference (<31 cm) as validated circumference-based proxies, in line with GLIM’s provision for their use where direct body composition methods (e.g., DXA, BIA) were unavailable. Etiologic criteria were: (4) reduced food intake, defined as estimated dietary energy intake (Intake24, an online 24 h recall diary [45]) at ≤75% of measured resting energy expenditure [46] (Indirect Calorimetry, GEMNutrition [47], as previously described [48]); and (5) disease burden or the presence of inflammation. The presence of MND, a chronic progressive disease, satisfies the etiological criteria required for this cohort [46]. Severity (moderate or severe) was graded from the phenotypic criterion met.

#### 2.5.2. Modified ESPEN Criteria

Participants were classified as being at risk of malnutrition if they had either: (1) a body mass index (BMI) < 18.5 kg/m^2^; or (2) unintentional weight loss > 10% from premorbid body weight (we did not apply ESPEN’s alternative >5%-over-3-month sub-criterion, since our weight-change measure reflects cumulative change from premorbid body weight over each participant’s full disease duration at M0, rather than a specific 3-month interval). To enhance the sensitivity for identifying individuals at risk of malnutrition in our MND cohort, we adapted our protocol to include additional anthropometric and biochemical indicators: (3) a MUAC value of <23.5 cm; (4) a calf circumference of <31 cm; and (5) a reduction in serum biochemical analyte concentration below established reference ranges for healthy individuals, as defined by our local clinical laboratory. Participants meeting **any two** of the above outlined diagnostic criteria were classified as being at risk of malnutrition under this framework.

### 2.6. Statistical Analysis

Statistical analysis was conducted using IBM^®^ SPSS^®^ Statistics (IMB SPSS statistics for Macintosh, Version 31.0.0.0) and GraphPad Prism (GraphPad Software Inc, La Jolla, CA, USA, Version 10.6.1). Continuous variables were presented as mean [standard deviation (SD)] or median [interquartile range (IQR)]. Normality was assessed using the Shapiro–Wilk test.

Bivariate correlation analysis using Spearman’s rank correlation coefficient was conducted to assess the relationship between the concentration of biochemical analytes against assessments of disease severity and progression, anthropometric measures and indices of malnutrition. Bivariate correlation analysis was plotted with a linear regression line and 95% confidence intervals from the mean.

Intra-evaluator reliability for triplicate TSF measurements was assessed by calculating the relative technical error of measurement (TEM), with acceptability defined as <7.5% [22]. The mean [SD] value of both right and left limb measurements was calculated for each limb anthropometric indices (TSF, MUAC, AMA and CC). Paired Samples *t*-tests were conducted between left and right limb measurements at M0, to identify any significant asymmetrical changes in body composition.

The age of each participant at M0 was used for all equations and longitudinal analysis. Longitudinal data was analysed using Dunnett’s mixed-effects analysis [24] or the Wilcoxon test for nonparametric distributions of data [25]. Percentage change was calculated by setting measurements at M0 to 100%, with repeated measurements at M3, M6 and M9, expressed as a percentage of the M0 value. The threshold for statistical significance was *p* < 0.05 for all analyses.

## 3. Results

Twenty-four patients living with MND were recruited to this study. Twenty-two participants completed study assessments at M0, and blood samples were obtained from 19/22 (86.4%) participants at M0. Demographic, clinical and anthropometric assessments for the cohort who provided blood samples at M0 (*n* = 19) are shown in Table 1.

### 3.1. Assessment of Inflammatory State

The concentrations of routine inflammatory markers (ESR, white cell count, platelets, lymphocytes, fibrinogen, CRP) measured at M0 are presented in Table 2. An increase in at least one inflammatory parameter relative to our local clinical laboratory’s reference ranges was found in 9/19 (47.4%) of participants (seven males, two females). The most commonly elevated inflammatory marker was fibrinogen, raised in 8/19 (42.1%) of study participants. Platelets and lymphocyte cell count were not elevated in any participants.

The study cohort was classified into two groups: those with at least one elevated marker of inflammation (“inflammatory group”, *n* = 9), and those without any elevated markers of inflammation (“non-inflammatory group”, *n* = 10) (Table 3). M0 comparisons revealed that BMI was higher (*p* = 0.04), whereas ΔALSFRS-R (*p* = 0.06) and creatinine (*p* = 0.07) were both lower in the inflammatory group. Participants with confirmed inflammation at M0 (*n* = 9) were removed from further analyses.

The M0 serum analyte concentrations of the non-inflammatory group were compared to the standard healthy clinical reference ranges, where available (Table 4). Retinol-binding protein was elevated above the upper reference limits in 90% (9/10) of participants.

### 3.2. Relationship of Serum Biochemical Analytes with Demographic, Clinical and Anthropometric Data at M0

**Figure 1:**
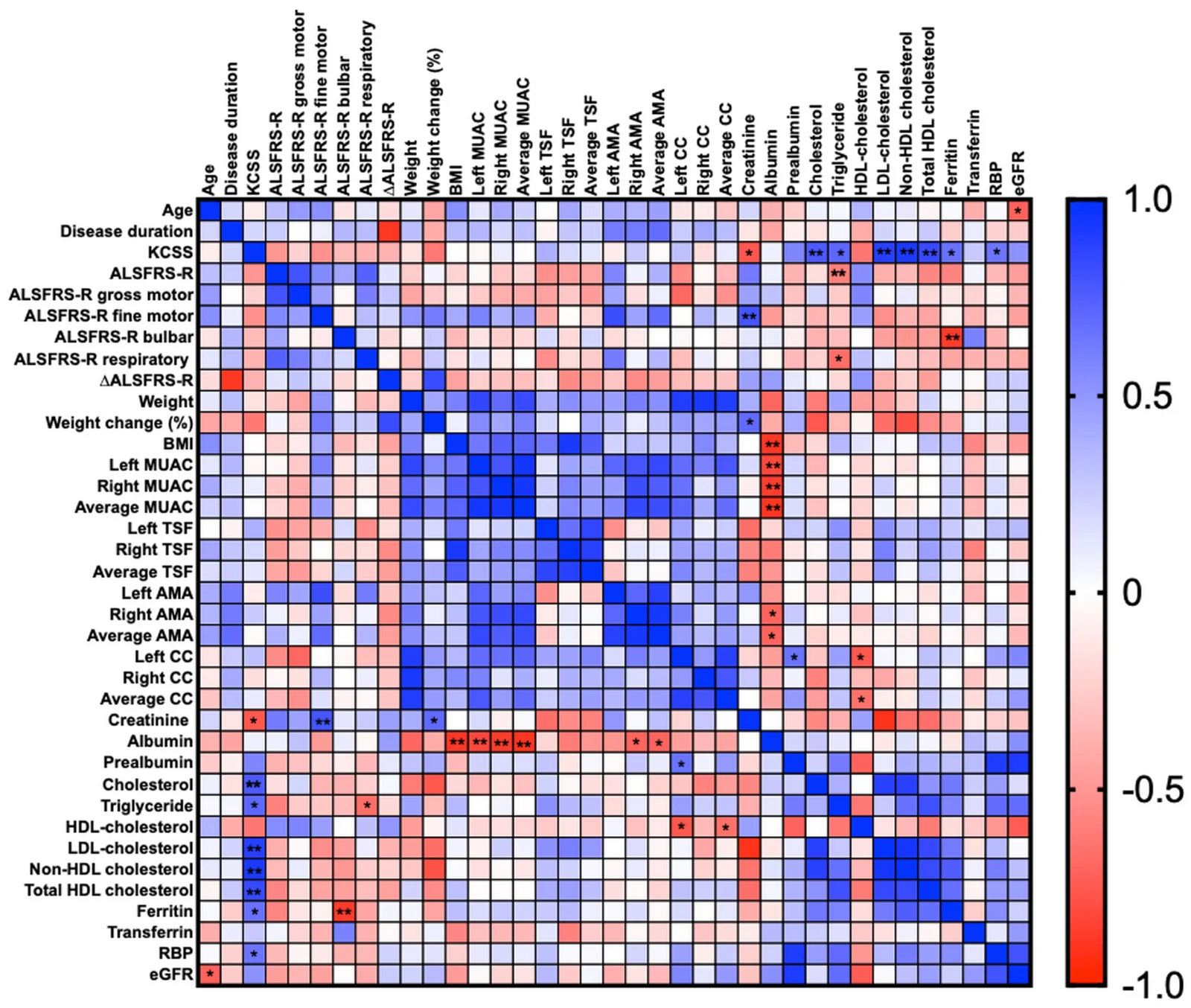
Correlation matrix of serum biochemical analytes with demographic, clinical and anthropometric data from a sub-cohort of participants without inflammation at M0. Correlation analysis was conducted using Spearman’s rank-order correlation analysis for nonparametric data. Cells are coloured according to the correlation coefficient using a diverging colour scale: deep blue indicates strong negative correlations (−1.0), white indicates near-zero correlation (0.0), and deep red indicates strong positive correlations (+1.0). Colour intensity increases with the magnitude of the correlation. Significance was observed at *p* < 0.05, indicated by * (*p* < 0.05) or ** (*p* < 0.01). ALSFRS-R: amyotrophic lateral sclerosis functional rating scale—revised; AMA: arm muscle area; BMI: body mass index; CC: calf circumference; MUAC: mid-upper arm circumference; TSF: triceps skinfold thickness; ∆ALSFRS-R: change in the amyotrophic lateral sclerosis functional rating scale—revised.

Bivariate correlation analysis was conducted to explore the relationship between the serum biochemical analytes and the clinical and anthropometric parameters of the non-inflammatory sub-cohort at M0 (Figure 1, Supplementary Table S1). The King’s College Staging System demonstrated strong positive correlations with the lipid profile assessments (cholesterol: r = 0.77, *p* = 0.01; triglyceride: r = 0.71, *p* = 0.022; LDL cholesterol: r = 0.89, *p* = 0.003; non-HDL cholesterol: r = 0.92, *p* < 0.001; total HDL cholesterol: r = 0.84, *p* = 0.002), ferritin (r = 0.66, *p* = 0.037) and RBP (r = 0.65, *p* = 0.042), but negatively correlated against creatinine (r = −0.76, *p* = 0.011). No correlations were observed between the serum biochemical analytes and the total ALSFRS-R score. However, negative correlations were observed between ferritin and the ALSFRS-R bulbar subscore (r = −0.87, *p* = 0.001); and triglyceride and the ALSFRS-R respiratory subscore (r = −0.67, *p* = 0.035). Conversely, creatinine positively correlated against the ALSFRS-R fine motor subscore (r = 0.79, *p* = 0.007).

In terms of body composition parameters, creatinine was positively associated with percentage weight change from ‘premorbid body weight’ (r = 0.83, *p* = 0.042). Albumin was negatively associated with BMI (r = −0.88, *p* = 0.004), MUAC (r = −0.82–−0.90, *p* < 0.001–0.004) and AMA (r = −0.71, *p* = 0.033). Prealbumin was positively correlated against left CC (r = 0.65 m *p* = 0.041), whilst HDL cholesterol was negatively correlated against left (r = −0.75, *p* = 0.012) and average CC (r = −0.66, *p* = 0.039).

### 3.3. Application of GLIM and Modified ESPEN Criteria for the Identification of Malnutrition

Using the modified ESPEN-based framework [44], one participant in our non-inflammatory analytical sub-cohort (1/10, 10%) was identified as being at risk of malnutrition at M0 (Table 5, participant A). Applying the GLIM criteria [43], two participants (2/10, 20%) met at least one phenotypic criterion for malnutrition at M0: participant A (severe low BMI, 17.7 kg/m^2^; reduced muscle mass, MUAC 22.25 cm) and participant J (moderate low BMI, 19.2 kg/m^2^) (Table 5). By default, the presence of MND itself satisfies GLIM’s “chronic disease-related” etiologic burden in all participants. However, no participants demonstrated a reduced dietary intake relative to measured resting energy expenditure, and the presence of inflammation could not be met by any participant in this sub-cohort, since sub-cohort membership itself required the absence of any elevated inflammatory marker (Table 2).

The two frameworks therefore identify an overlapping but not identical set of participants: both retain participant A, but GLIM’s age-adjusted moderate-BMI threshold (<20 kg/m^2^ for age < 70 years) additionally identifies participant J, who falls beyond our modified ESPEN framework’s single < 18.5 kg/m^2^ cut-off, but is within GLIM’s moderate range.

### 3.4. Longitudinal Changes in Biochemical Analytes

**Figure 2:**
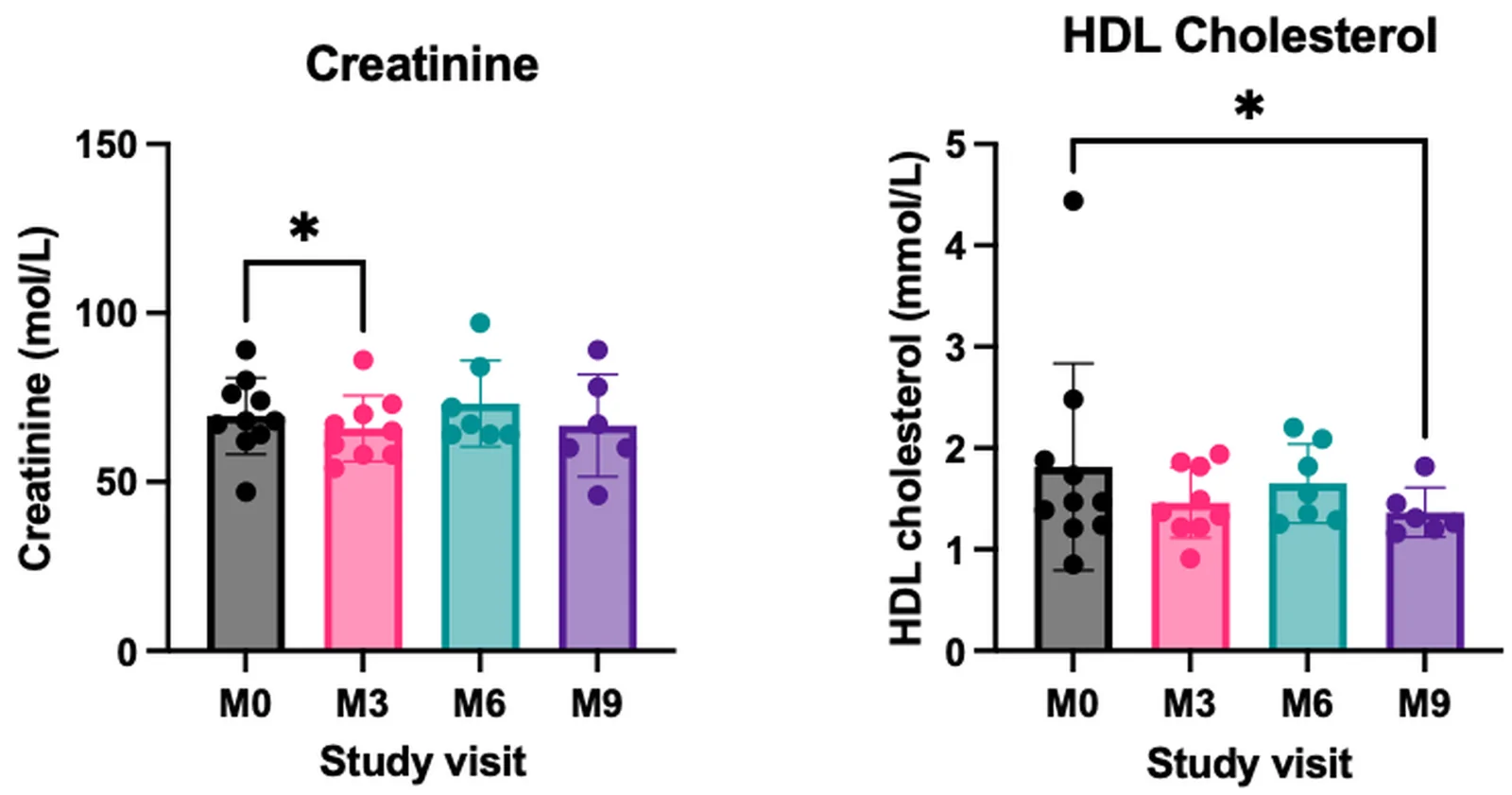
Longitudinal analysis of serum creatinine (**left**) and HDL cholesterol (**right**) from the subgroup without inflammation. Data is grouped for each study visit, presented as mean ± SD. M0 *n* = 10; M3 *n* = 9; M6 *n* = 7; M9 *n* = 6. Changes in longitudinal data were analysed for significance using Wilcoxon matched-pairs signed rank test for non-normally distributed data. Significance was observed at *p* < 0.05 (*) Creatinine significantly declined between month 0 and month 3 (*p* = 0.02). HDL cholesterol significantly declined between month 0 and month 9 (*p* = 0.03). M0–9: months 0–9; SD: standard deviation.

Longitudinal analysis of the serum biochemical analytes was conducted for participants in the non-inflammatory sub-cohort (M0: *n* = 10; M3: *n* = 9; M6: *n* = 7; M9: *n* = 6). A decline in creatinine levels was observed for M0 and M3 (*p* = 0.02, *n* = 9), alongside a long-term reduction in HDL cholesterol between M0 and M9 (*p* = 0.03, *n* = 6) (Figure 2, Supplementary Table S2). These findings are comparable to longitudinal assessments regardless of inflammatory state, whereby reductions in serum creatinine were observed between M0 and M3 (*p* = 0.02, *n* = 15) and between M0 and M9 (*p* = 0.03, *n* = 10) (Supplementary Table S3). These additional significant observations may be attributed to the larger sample size over the nine-month study period, regardless of inflammatory state. HDL cholesterol was also observed to decline between M0 and M9 for the entire study cohort, regardless of inflammation state (*p* = 0.002, *n* = 10) (Supplementary Table S3). No significant changes were observed longitudinally in any of the biochemical analytes for the inflammatory sub-cohort (Supplementary Table S4).

## 4. Discussion

### 4.1. Inflammatory State at Baseline

Our findings of modest and heterogeneous elevations in systemic inflammatory markers—most notably fibrinogen in nearly half of participants (42.1%)—align with the broader literature demonstrating inconsistent but recurrent evidence of low-grade peripheral inflammation in subsets of people living with MND [49,50,51,52,53]. These inflammatory abnormalities are clinically relevant because routine nutritional biomarkers, such as albumin, prealbumin, transferrin, and creatinine, are all influenced by acute-phase responses, thus confounding the interpretation of malnutrition in MND, where true nutritional deficits frequently coexist with inflammation-induced biochemical changes [54,55,56,57]. In keeping with this, our study demonstrates that participants with elevated inflammatory markers showed trends toward altered creatinine concentrations and differences in BMI, justifying their exclusion from subsequent nutritional analyses.

### 4.2. Nutritional and Metabolic Profiles

In our non-inflammatory sub-cohort (*n* = 10), serum creatinine correlated strongly with muscle-related functional subscores and declined between M0 and M3, although a non-significant increase in serum creatinine between M0 and M6 should also be noted (Figure 2, left). Among participants with available paired M0–M6 data (*n* = 7), five experienced declines and two experienced increases. The median change of −4 units indicates overall relative stability; nevertheless, with substantial inter-individual variability. Falling creatinine has been shown to reflect ongoing muscle wasting and predict faster functional decline and shorter survival [58]. Thus, the decline in creatinine observed in our cohort is biologically plausible and aligns closely with the established MND literature identifying creatinine as a practical, inexpensive, and clinically informative biomarker of muscle mass depletion and early nutritional deterioration. It should be acknowledged, however, that creatinine decline in MND is not exclusive to nutritional wasting: progressive neurogenic muscle denervation will also reduce creatinine independent of nutritional intake, as even a well-nourished patient with MND will lose creatinine in proportion to their muscle loss. Creatinine should therefore be interpreted as a marker of total muscle mass loss rather than nutritional wasting per se; it should ideally be paired with anthropometric indices of body composition and nutritional intake data, to contextualise the relative contributions of denervation and malnutrition.

Because serum creatinine is largely determined by skeletal muscle mass [58], its interpretation may also differ according to the MND phenotype. This may be particularly relevant to primary lateral sclerosis (PLS), in which upper motor neuron-predominant pathology can give rise to prominent bulbar dysfunction and dysphagia, while lower motor neuron involvement—and, by extension, muscle bulk—remains comparatively preserved, particularly in the earlier stages of disease [59]. It is therefore plausible that creatinine could remain relatively preserved in PLS despite clinically meaningful nutritional compromise, which would have important implications for how this biomarker is interpreted across different MND phenotypes. However, our non-inflammatory sub-cohort included only a single participant with PLS, precluding any meaningful comparison of creatinine trajectories across phenotypes. This observation should therefore be regarded as hypothesis-generating rather than conclusive, and warrants dedicated investigation in phenotype-balanced cohorts.

### 4.3. Lipid Profile and Metabolic Correlates

The positive correlations observed between the King’s College Staging System and lipid parameters in our cohort support accumulating evidence that altered lipid metabolism represents a key metabolic feature of MND, with important implications for nutritional assessment. Elevated serum lipids, particularly total cholesterol and triglycerides, may reflect adaptive responses to increased energy expenditure or hypermetabolism, potentially serving as compensatory energy reserves and being associated with improved survival in some cohorts [60]. However, considerable heterogeneity exists across the MND literature, underscoring the importance of interpreting lipid fractions individually rather than collectively. For example, while higher triglyceride levels among MND patients have been associated with lower mortality, higher HDL or LDL cholesterol levels have paradoxically been linked to increased mortality in some populations [61]. These findings suggest that lipid profiles in MND reflect complex interactions between energy balance, metabolic stress, and disease progression, highlighting the need for nuanced nutritional evaluation in which lipid markers are interpreted within the broader metabolic and clinical context, rather than as isolated indicators of nutritional intake or deficiency.

Longitudinal changes in lipid parameters further emphasise the importance of dynamic nutritional monitoring in MND. The decline in HDL cholesterol observed between M0 and M9 aligns with longitudinal and meta-analytic evidence demonstrating that lipid profiles evolve with disease progression, often reflecting increasing metabolic dysregulation, systemic inflammation, or worsening nutritional status [62,63,64,65]. Notably, higher HDL cholesterol at diagnosis has been associated with poorer survival in hypermetabolic patients, suggesting that elevated HDL may represent metabolic stress rather than a protective lipid phenotype [66]. The negative correlation between serum triglycerides and respiratory function reported in the MND literature further supports the link between energy availability and functional decline, particularly in energy-demanding systems such as respiratory musculature [61].

Collectively, these findings indicate that disrupted lipid metabolism in MND is multifaceted, with triglycerides potentially acting as an energy buffer, cholesterol contributing to structural and metabolic reserves, and HDL dynamics reflecting systemic physiological stress. Accordingly, nutritional assessment in MND should incorporate longitudinal lipid profiling alongside functional and metabolic measures. In practice, serial lipid tracking may help distinguish patients in whom nutritional decline is accompanied by falling triglycerides and lipid reserves (suggesting inadequate caloric intake) from those with elevated or stable lipids driven by hypermetabolism or disease-related metabolic reprogramming (suggesting intact energy availability despite functional decline). This distinction has direct relevance to the timing and composition of personalised nutritional interventions—for example, guiding the intensity of caloric supplementation or the use of high-fat dietary strategies in hypermetabolic individuals [66,67].

### 4.4. Ferritin, Bulbar Features and Survival

We observed a negative correlation between ferritin and the ALSFRS-R bulbar subscore at M0. However, the wider literature is heterogeneous: while several studies report that higher ferritin predicts faster progression or reduced survival [68,69,70], others report only modest or no associations after adjusting for key confounders (e.g., inflammation, nutritional status, disease duration) [71]. Some analyses suggest that serum ferritin’s prognostic value may differ by phenotype, sex, or geographic cohort [72,73]. Although our ferritin–bulbar association is consistent with previous observations, the causal meaning (e.g., whether driven by iron loading, inflammation, or catabolism) remains undetermined.

### 4.5. Retinol-Binding Protein and Nutritional Markers

Retinol-binding protein was elevated (relative to healthy reference ranges) in nearly all participants (90%) in our non-inflammatory sub-cohort. Published MND data on retinol-binding protein is limited and inconsistent. The retinol-binding protein elevation observed within our cohort contrasts with a prior report indicating a lower median value of RBP4 (a retinol-binding protein isoform) in MND cases relative to controls [74]. This discrepancy may reflect differences in the measured isoform, assay/reference ranges, small-sample effects, or population clinical heterogeneity. This highlights the need for replication and assay harmonisation within the field of nutritional biomarker in MND [74].

### 4.6. Anthropometric and Functional Associations

We observed unexpected negative correlations between albumin and BMI, MUAC and AMA at M0. Several factors could plausibly explain this: (i) albumin is an acute-phase reactant which may be influenced by inflammation and hydration status; (ii) BMI in MND may reflect preserved fat mass despite muscle loss; and (iii) restricted sample sizes inherent to pilot studies may introduce spurious correlations. The MND nutritional literature evidence supports that lower albumin and lower BMI tend to link with shorter survival [75,76]; hence, our inverse correlations likely reflect these confounding or cohort-specific features, rather than a true physiological reversal.

In contrast, creatinine demonstrated expected relationships with functional status, correlating positively with fine motor ALSFRS-R subscores, and with premorbid weight change. This is consistent with reports in the MND literature linking lower serum creatinine levels to rapid functional decline and shorter survival, with creatinine displaying a positive correlation with ALSFRS-R scores and inversely with decline rate [77].

### 4.7. Identifying Malnutrition

#### 4.7.1. Use of the Modified ESPEN and GLIM Comparator Frameworks

Applying the GLIM criteria alongside our modified ESPEN-based framework illustrates that the two are not interchangeable, at least for our cohort. GLIM’s age-adjusted, moderate low BMI threshold (<20 kg/m^2^ for age < 70 years) identified an additional participant (J) beyond those flagged by our modified ESPEN framework’s single <18.5 kg/m^2^ cut-off, indicating that GLIM may be more sensitive to phenotypic malnutrition risk. More fundamentally, the inclusion of the GLIM’s etiologic criterion is subjective to personal interpretation and validation; in this situation, the decision to consider the presence of MND as satisfactory was made a priori—as per recommendation by Barone et al., (2023) [46]. Our non-inflammatory sub-cohort is, by design, defined by the absence of any elevated inflammatory marker, so the inflammation etiologic pathway is structurally unavailable within it, and although this study collected dietary intake and measured resting energy expenditure data, neither participant with a positive phenotypic finding showed reduced intake relative to requirement. This could potentially signify a structural tension between GLIM’s inflammation-inclusive design and a sub-cohort deliberately stratified to remove that same signal, rather than as evidence that malnutrition risk is absent in these participants; the phenotypic findings (reduced BMI and muscle mass) suggest otherwise. Future work applying GLIM to MND cohorts that do not require this stratification, where the inflammation pathway remains available, may therefore yield a more complete picture (as previously demonstrated for GLIM-defined malnutrition and survival in ALS [78]), and we recommend GLIM be applied to the full, unstratified cohort alongside any inflammation-based sub-analysis.

#### 4.7.2. Biochemical Markers of Malnutrition

A distinct subset of participants (3/10, 30%) in our non-inflammatory sub-cohort presented biochemical indications of malnutrition (low transferrin or low creatinine) (Table 5). This aligns with the estimated prevalence of malnutrition in MND (16–55%) that can be attributed to dysphagia, hypermetabolism, and neurogenic muscle atrophy. Regardless of aetiology (i.e., disuse, denervation, or catabolic muscle wasting from nutritional deficit), progressive muscle atrophy results in reduced protein turnover and a fall in transferrin and creatinine. These findings support that routine, minimally invasive laboratory markers (transferrin, creatinine, albumin, prealbumin) have important practical utility in nutritional surveillance. Serial measurement of these markers can possibly flag progressive nutritional deterioration even in patients whose BMI remains within normal ranges, as was the case in our cohort, thus prompting proactive clinical review, dietary optimisation, or earlier gastrostomy discussions when interpreted alongside inflammatory markers and anthropometric assessment.

### 4.8. Strengths and Limitations

The main strength of this proof-of-concept pilot study lies in its prospective, longitudinal design and the robust assessment protocols. Detailed biochemical, clinical, and anthropometric data were collected, enabling multidimensional assessment of nutritional, metabolic, and inflammatory status. Stratification by inflammatory status reduced potential confounding when interpreting hepatic and nutritional biomarkers. The use of subscore-level ALSFRS-R data improved the granularity of functional correlations relative to assessments of nutritional status. Longitudinal sampling allowed the detection of biochemical changes over time, particularly reductions in creatinine and HDL cholesterol.

The primary limitation is the restricted sample size remaining after strict inflammatory exclusions, alongside missing data for select anthropometric variables, and high longitudinal attrition (attrition of 36.4%: 22 participants at M0 dropping to 14 participants at M9). This attrition rate is reflective of disease-related mortality and functional burden, which is common in prospective MND cohorts. Second, data were collected from patients recruited from a single specialised MND care centre, reducing immediate generalisability and increasing the likelihood that the observed correlations are cohort-specific. Third, only routine clinical inflammatory and biochemical markers were assessed; more sensitive markers of neuroinflammation or neuronal injury (e.g., cytokines, NfL) were beyond the scope of this study. Lastly, whilst restricting downstream analyses to non-inflammatory participants likely reduced confounding, it may have excluded clinically relevant phenotypes and/or inherently reduced statistical power. In particular, the small number of participants within three distinct MND phenotypes in the non-inflammatory sub-cohort (ALS: 7/10; PMA: 2/10; PLS: 1/10) precluded any meaningful statistical comparison between MND phenotypes. Such comparisons are of considerable clinical interest, since nutritional impairment would be expected to differ according to phenotype; for example, participants with prominent bulbar involvement might be expected to exhibit more marked nutritional compromise because of dysphagia [6,7,8,9,10,11,12,13,14,15,16,17,18,19,20,21,22,23,24,25,26,27,28,29,30,31,32,33,34,35,36,37,38,39,40,41,42,43,44,45,46,47,48,49,50,51,52,53,54,55,56,57,58,59,60,61,62,63,64,65,66,67,68,69,70,71,72,73,74,75,76,77,78,79], whereas those with predominantly lower motor neuron-driven phenotypes such as PMA, or with limb-onset ALS, may follow a different trajectory. This represents an important limitation of the present study, and phenotype-specific nutritional trajectories should be examined explicitly in future, adequately powered cohorts. We therefore acknowledge the potential trade-off between internal validity and sample size [80,81]. We recommend that future studies prioritise the recruitment of larger, multicentre cohorts with adequate representation across MND phenotypes, so that robust, phenotype-specific conclusions regarding the clinical utility of these biomarkers can be drawn.

## 5. Summary and Key Findings

Our prospective longitudinal data support several key themes within the MND literature: (1) our study demonstrated that low-grade systemic inflammation is present in almost 50% of participants with MND, supporting the need for inflammatory assessment in biochemical analyses; (2) in the non-inflammatory participants, serum creatinine showed early and progressive decline and correlated with muscle-related functional measures, suggesting it may be used as a sensitive longitudinal marker of muscle wasting; (3) dynamic longitudinal changes in lipid fractions—particularly declining HDL cholesterol—reflected evolving metabolic stress rather than static nutritional status; and (4) biochemical markers routinely available in clinical practice, such as albumin, prealbumin, creatinine, and transferrin, hold clear value for nutritional surveillance, provided that the potential presence of inflammation is considered during interpretation.

This study identified a high prevalence of elevated serum retinol-binding protein in non-inflammatory MND, suggesting altered retinoid or muscle-associated metabolic signalling and highlighting a novel avenue for further investigation. The combined use of biochemical, anthropometric, and functional measures highlights that biochemical evidence of malnutrition can be detected even when BMI remains within normal ranges, underscoring the clinical inadequacy of relying on BMI alone. We propose that standard clinical practice should transition toward a composite monitoring approach. Integrating serial measurements of serum creatinine, transferrin, and lipid fractions alongside longitudinal MUAC and percentage weight change could provide an early-warning signal for nutritional deterioration. This would enable personalised, timely dietary optimisation or consideration of interventions, such as enteral feeding, before overt functional decline occurs.

## 6. Future Directions

Future studies should deploy larger, multicentre validation cohorts using harmonised assay platforms and repeated longitudinal sampling to improve the precision and generalisability of biomarker trajectories in MND. Integration of routine serum analytes with advanced molecular biomarkers (e.g., cytokines, neurofilaments, metabolomic and proteomic profiles) may allow for the early detection of subtle inflammatory and metabolic alterations across the disease course. Stratification by phenotype, inflammatory state and metabolic profile could further identify biologically distinct patient subgroups with divergent biochemical patterns and clinical trajectories. In particular, larger cohorts with adequate representation of less common phenotypes such as PMA and PLS are needed to test hypotheses generated by this pilot study, including whether the relationship between serum creatinine and nutritional status differs according to the pattern of upper versus lower motor neuron involvement. Ultimately, the development and validation of disease-specific composite biomarker algorithms—integrating anthropometry, functional measures and biochemical indices—may support the early identification of individuals at imminent nutritional risk and guide personalised, and proactive therapeutic intervention.

## Figures and Tables

**Table 1:** Demographic, clinical and nutritional assessments for the cohort at M0 (*n* = 19).

	*n* = 19
Sex	
Male	15/19 (78.9%)
Female	4/19 (21.1%)
**Age, years**	62.95 ± 11.33 61 (56–72)
**Phenotype**	
ALS	13/19 (68.4%)
PMA	4/19 (21.1%)
PLS	1/19 (5.3%)
Unspecified	1/19 (5.3%)
**Site of Onset**	
Bulbar	3/19 (15.8%)
Upper limb	5/19 (26.3%)
Lower limb	7/19 (36.8%)
Respiratory	2/19 (10.5%)
Mixed	2/19 (10.5%)
**Disease duration (months)**	47.74 ± 49.91 26 (24–49)
**King’s College Staging System**	
Stage 1	3/19 (15.8%)
Stage 2	3/19 (15.8%)
Stage 3	6/19 (31.6%)
Stage 4	7/19 (36.8%)
**ALSFRS-R (/48)**	
Total Score	33.89 ± 6.32 32 (29–41)
Bulbar subscale	9.47 ± 2.65 10 (8–12)
Fine motor subscale	8.05 ± 2.39 8 (7–10)
Gross motor subscale	7.37 ± 2.56 8 (6–9)
Respiratory subscale	9.00 ± 3.54 11 (7–12)
ΔALSFRS-R	0.58 ± 0.50 0.53 (0.12–0.75)
**Gastrostomy**	
Present	4/19 (21.05%)
Not present	15/19 (78.95%)
**Ventilation**	
No respiratory support	13/19 (68.4%)
Intermittent use	-
Overnight	5/19 (26.3%)
Overnight and intermittent throughout the day	1/19 (5.3%)
24 h use	-
**Anthropometric Measurement or Indices**	
Weight (kg)	79.01 ± 18.52 83.40 (64.45–91.00)
Percentage weight loss (%)	−5.11 ± 7.57 −6.07 (−8.57–−2.63)
Body mass index (kg/m^2^)	26.39 ± 4.87 26.00 (25.50–30.35)
Mid-upper arm circumference (cm)	29.73 ± 3.31 30.15 (27.35–32.20)
Triceps skinfold thickness (mm)	13.15 ± 6.66 14.09 (7.29–17.65)
Arm Muscle Area (cm^2^)	52.90 ± 13.76 47.56 (44.56–61.54)
Calf circumference (cm)	37.35 ± 3.70 36.80 (34.65–39.85)

The number of participants per assessment is presented as *n*/N (percentage of population, %). Continuous data is presented as mean ± SD and median (IQR). ALS: amyotrophic lateral sclerosis; ALSFRS-R: amyotrophic lateral sclerosis functional rating scale—revised; IQR: interquartile range; kcal/day: kilocalories per day; PLS: primary lateral sclerosis; PMA: progressive muscular atrophy; ∆ALSFRS-R: change in functional score.

**Table 2:** Markers of inflammation for the study cohort at M0 (*n* = 19).

Biosample	Inflammatory Marker	Mean ± SD Median (IQR)	Reference Ranges	Elevated *n*/N (%)
**Whole blood**	Erythrocyte sedimentation rate (mm/hr)	10.79 ± 10.03 6.00 (5.00–20.00)	Male: 1–10 Female: 1–15	5/19 (26.32)
	White cell count (×10^9^/L)	6.49 ± 1.99 6.10 (5.40–7.10)	3.5–9.5	2/19 (10.53)
	Platelets (×10^9^/L)	248.05 ± 69.34 241.00 (184.00–310.00)	150–400	0/19 (0.00)
	Lymphocytes (×10^9^/L)	1.46 ± 0.52 1.26 (1.06–1.92)	1.0–3.0	0/19 (0.00)
**Plasma**	Fibrinogen (g/L)	3.81 ± 0.89 3.90 (3.20–4.30)	2.0–4.0	8/19 (42.11)
**Serum**	C-Reactive Protein (mg/mL)	3.19 ± 6.87 1.60 (0.80–2.00)	0.00–5.00	2/19 (10.53)

Data is presented as mean ± one standard deviation and median (IQR). The number of participants with elevated inflammatory marker concentrations is indicated as *n*/N (%). IQR: interquartile range; SD: standard deviation.

**Table 3:** Comparisons of demographic, clinical and serum analyte parameters between inflammatory vs. non-inflammatory groups.

	Non-Inflammatory Group *n* = 10	Inflammatory Group *n* = 9	*p*-Value
**Phenotype**	ALS: 7/10 PMA: 2/10 PLS: 1/10 Unspecified: -	ALS: 6/9 PMA: 2/9 PLS: - Unspecified: 1/9	-
**Site of onset**	Limb: 6/10 Respiratory: - Bulbar: 3/10 Mixed: 1/10	Limb: 6/9 Respiratory: 2/9 Bulbar: - Mixed: 1/9	-
**Age (years)**	60.60 ± 12.13 60.50 (54.00–69)	65.56 ± 10.43 61.00 (57.00–75.50)	0.35
**Disease duration (months)**	44.90 ± 46.21 28.00 (23.50–49.50)	50.89 ± 56.39 26.00 (19.00–68.50)	0.81
**ALSFRS-R total score (/48)**	35.00 ± 6.15 34.00 (29.00–41.50)	32.67 ± 6.65 30.00 (29.00–37.50)	0.44
**Δ** **ALSFRS-R**	1.22 ± 0.66 1.18 (0.67–1.72)	0.65 ± 0.56 0.55 (0.18–1.13)	0.06
**King’s College Staging System**	2.70 ± 0.95 3.00 (2.00–3.25)	3.11 ± 1.27 4.00 (2.00–4.00)	0.44
**Weight**	N = 8 71.30 ± 13.28 69.50 (60.48–85.35)	N = 9 85.85 ± 20.48 89.20 (67.00–101.40)	0.10
**Percentage weight change (%)**	N = 6 −4.28 ± 3.01 −4.83 (−7.10–−1.77)	N = 9 −5.67 ± 9.68 −6.39 (−12.48–−2.22)	0.70
**BMI (kg/m^2^)**	N = 8 23.85 ± 4.05 25.15 (19.70–27.05)	N = 9 28.64 ± 4.57 29.10 (24.25–32.70)	**0.04**
**Creatinine (mol/L)**	69.50 ± 11.34 68.00 (63.50–77.00)	63.44 ± 22.93 56.00 (50.50–66.00)	0.07
**Albumin (g/L)**	45.90 ± 1.79 45.50 (44.75–48.00)	47.11 ± 2.67 46.00 (45.50–48.50)	0.27
**Prealbumin (g/L)**	0.27 ± 0.05 0.27 (0.23–0.30)	0.27 ± 0.05 0.28 (0.24–0.31)	0.92
**Cholesterol (mmol/L)**	5.33 ± 0.86 5.20 (4.45–5.78)	4.69 ± 0.97 4.90 (4.05–5.35)	0.23
**Triglyceride (mmol/L)**	1.49 ± 0.68 1.50 (0.95–1.95)	1.50 ± 0.56 1.50 (1.00–1.85)	0.97
**HDL cholesterol (mmol/L)**	1.82 ± 1.02 1.47 (1.23–2.03)	1.52 ± 0.48 1.61 (1.05–1.93)	0.95
**LDL cholesterol (mmol/L)**	2.90 ± 0.86 2.85 (2.20–3.30)	2.47 ± 0.68 2.40 (2.05–3.00)	0.27
**Non-HDL cholesterol (mmol/L)**	3.71 ± 1.03 3.70 (2.80–4.53)	3.18 ± 0.66 3.40 (2.50–3.65)	0.20
**Total HDL cholesterol ratio (mmol/L)**	3.70 ± 1.20 3.70 (2.58–4.78)	3.29 ± 0.89 3.10 (2.70–3.60)	0.62
**Ferritin (µg/L)**	243.20 ± 76.62 240.00 (179.75–305.50)	261.11 ± 149.97 226.00 (151.50–412.50)	0.75
**Transferrin (g/L)**	2.27 ± 0.20 2.31 (2.07–2.49)	2.52 ± 0.39 2.62 (2.13–2.85)	0.11
**Retinol-binding protein (mg/L)**	50.80 ± 12.25 45.00 (40.75–61.75)	56.56 ± 12.16 58.00 (46.00–65.00)	0.32

Data presented as mean ± one standard deviation and median (IQR). Normally distributed data was analysed using Welch’s *t*-test; non-normally distributed data was analysed using Mann–Whitney test. Significance at *p* < 0.05 level. ALSFRS-R: amyotrophic lateral sclerosis functional rating scale—revised; BMI: body mass index; IQR: interquartile range; kcal/day: kilocalories per day; ∆ALSFRS-R: change in functional score.

**Table 4:** Serum analyte concentrations at enrolment (M0) for the participants without inflammation (*n* = 10).

	Reference Ranges	M0	Decreased	Elevated
**Creatinine (mol/L)**	Male: 62–106 Female: 44–80	N = 10 69.50 ± 11.34 68.00 (63.50–77.00)	2/10	-
**Albumin (g/L)**	35–50	N = 10 45.90 ± 1.79 45.50 (44.75–48.00)	-	-
**Prealbumin (g/L)**	Male: 0.2–0.5 Female: 0.1–0.4	N = 10 0.27 ± 0.05 0.27 (0.23–0.30)	-	-
**Cholesterol (mmol/L)**		N = 10 5.22 ± 0.86 5.20 (4.45–5.78)		
**Triglyceride (mmol/L)**		N = 10 1.49 ± 0.68 1.50 (0.95–1.95)		
**HDL cholesterol (mmol/L)**		N = 10 1.82 ± 1.02 1.47 (1.23–2.03)		
**LDL cholesterol (mmol/L)**		N = 8 2.90 ± 0.86 2.85 (2.20–3.30)		
**Non-HDL cholesterol (mmol/L)**		N = 10 3.71 ± 1.03 3.70 (2.80–4.53)		
**Total HDL cholesterol ratio (mmol/L)**		N = 10 3.71 ± 1.20 3.70 (2.58–4.78)		
**Ferritin (** **µ** **g/L)**	Males: 30–400 Females <60 yr: 15–150 Females >60 yr: 30–400	N = 10 243.20 ± 76.62 240.00 (179.75–305.50)	-	1/10
**Transferrin (g/L)**	2.0–3.2	N = 10 2.27 ± 0.20 2.31 (2.07–2.49)	1/10	-
**Retinol-binding protein (mg/L)**	20–40	N = 10 50.80 ± 12.25 42.00 (40.75–61.75)	-	9/10
**eGFR EPI**		N = 9 86.44 ± 6.64 90.00 (84.50–90.00)		

Concentrations were compared to reference ranges provided by the Northern General Hospital Medical Laboratory, where provided. Data presented as mean ± one standard deviation and median (IQR). The number of participants with decreased or elevated serum analyte concentrations is indicated as *n*/N.

**Table 5:** Markers for the risk of malnutrition for the sub-cohort without inflammation at enrolment (M0), *n* = 10.

Pt ID	Sex	Age	Weight Change (%)	BMI (kg/m^2^)	MUAC Avg (cm)	CC Avg (cm)	Creatinine (mol/L)	Albumin (g/L)	Prealbumin (g/L)	Ferritin (µg/L)	Transferrin (g/L)	RBP (mg/L)	Dietary Intake (% of Measured REE)	At Risk—Modified ESPEN	At Risk—GLIM *	GLIM Phenotypic Criteria Met
A	M	37	−4.81	17.70	22.25	32.75	68.00	48.00	0.23	149.00	2.51	40.00	148%	Yes	Yes (severe)	Low BMI (severe); reduced muscle mass
B	M	56	0.81	26.00	32.35	39.35	80.00	45.00	0.29	295.00	2.09	55.00	156%	No	No	None
C	M	80	-	27.40	30.15	36.45	89.00	44.00	0.22	130.00	2.34	38.00	140%	No	No	None (weight loss not assessable)
D	F	72	−7.09	29.00	27.35	34.65	64.00	45.00	0.22	352.00	1.95	44.00	112%	No	No	None
E	M	48	-	26.00	26.75	39.10	62.00	47.00	0.28	208.00	2.27	44.00	110%	No	No	None (weight loss not assessable)
F	M	65	-	-	31.15	36.50	47.00	45.00	0.35	291.00	2.48	73.00	128%	No	Not assessable	Not assessable (BMI/weight loss missing)
G	F	59	-	-	32.40	37.10	67.00	43.00	0.24	267.00	2.21	41.00	83%	No	Not assessable	Not assessable (BMI/weight loss missing)
H	M	59	−7.14	21.20	26.20	35.55	68.00	48.00	0.29	337.00	2.5	60.00	165%	No	No	None
I	M	62	−2.63	24.30	29.25	36.80	74.00	46.00	0.33	213.00	2.35	67.00	150%	No	No	None
J	M	68	−4.84	19.20	26.35	34.70	76.00	48.00	0.26	190.00	2.02	46.00	179%	No	Yes (moderate)	Low BMI (moderate)

Malnutrition was indicated using both a modified ESPEN diagnostic criteria [44] and the GLIM criteria [43]. Under the modified ESPEN framework, participants were indicated to be at risk of malnutrition when presenting with reductions in any two of the following: percentage weight loss from premorbid body weight (>10%); BMI (<18.5 kg/m^2^); MUAC (<23.5 cm); calf circumference (<31 cm); and serum biochemical analytes below local hospital reference ranges (Table 4). Under GLIM, at least one phenotypic criterion (low BMI, weight loss beyond 6 months, or reduced muscle mass via MUAC/calf circumference) and at least one etiologic criterion (reduced food intake, ≤75% of measured resting energy expenditure; or disease burden/inflammation) were both required. * The etiological criterion was satisfied in all participants by the presence of MND. Data not collected is denoted by ‘–’. Parameters indicating a risk of malnutrition are highlighted in red, parameters above thresholds are indicated in green. Avg: average taken from left and right limb circumference measurements; BMI: body mass index; CC: calf circumference; ESPEN: European Society for Clinical Nutrition and Metabolism; F: female; GLIM: Global Leadership Initiative on Malnutrition M: male; MUAC: mid-upper arm circumference; Pt ID: participant ID; REE: resting energy expenditure; RBP: retinol-binding protein.

## Data Availability

To protect participant confidentiality, raw clinical data are not publicly deposited. The final anonymised dataset supporting the findings of this study may be made available from the corresponding author [T.S.] upon reasonable request, subject to meeting ethical and research governance requirements associated with this study.

## Supplementary Materials

The following supporting information can be downloaded at: https://www.mdpi.com/article/10.3390/nu18172913/s1, Table S1: Correlation of baseline serum biochemical analytes with demographic, clinical and anthropometric data from a sub-cohort of participants without inflammation.; Table S2: Longitudinal serum analyte concentrations for the group without inflammation; Table S3: Longitudinal serum analyte concentrations for the entire cohort; Table S4: Longitudinal serum analyte concentrations for the group with inflammation.

## Rights Retention Statement

For the purpose of open access, the author has applied a Creative Commons Attribution (CC BY) licence to any Author Accepted Manuscript version arising.

## Abbreviations

The following abbreviations are used in this manuscript:

AMA	Arm Muscle Area
ALS	Amyotrophic Lateral Sclerosis
ALSFRS-R	Amyotrophic Lateral Sclerosis Functional Rating Scale—Revised
BIA	Bioelectrical Impedance Analysis
BMI	Body Mass Index
CC	Calf Circumference
CRP	C-Reactive Protein
DXA	Dual-Energy X-Ray Absorptiometry
eGFR	Estimated Glomerular Filtration Rate
ESR	Erythrocyte Sedimentation Rate
HDL	High Density Lipoprotein
KCSS	King’s College Staging System
Kg/m^2^	Kilogrammes Per Metre Squared
LDL	Low Density Lipoprotein
M0-9	Month 0-9
MND	Motor Neuron Disease
MUAC	Mid-Upper Arm Circumference
NGH	Northern General Hospital
PLS	Primary Lateral Sclerosis
PMA	Progressive Muscular Atrophy
RBP	Retinol-Binding Protein
REE	Resting Energy Expenditure
TEM	Technical Error of Measurement
TSF	Triceps Skinfold Thickness
UoS	University of Sheffield
