# Supplementary material for: Serum Biomarkers for Nutritional Monitoring in Motor Neuron Disease: A Longitudinal Cohort Study
Source: Nutrients. 2026 Sep 4;18(17):2913. doi: 10.3390/nu18172913 (PMC13567484; doi:10.3390/nu18172913)
Supplement: Supplementary file 1 [file nutrients-18-02913-s001.zip › nutrients-4495048-supplementary.pdf]

Supplementary Table S1. **Correlation of baseline serum biochemical analytes with demographic, clinical and anthropometric data from a sub-cohort of participants without inflammation.**

		Creatinine (mol/L)	Albumin (g/L)	Prealbumin (g/L)	Cholesterol (mmol/L)	Triglyceride (mmol/L)	HDL cholesterol (mmol/L) †	LDL cholesterol (mmol/L)	Non-HDL cholesterol (mmol/L)	Total HDL-cholesterol (mmol/L)	Ferritin (µg/L)	Transferrin (g/L)	Retinol-binding protein (mg/L)	eGFR †
**Demographic and Clinical**														
Age (years)	*r*	0.204	-0.38	-0.254	0.076	0.043	0.36	0.06	0.113	-0.061	0.024	-0.389	0.018	**-.715***
	p	0.571	0.279	0.479	0.834	0.906	0.307	0.887	0.756	0.867	0.947	0.266	0.96	**0.03**
	n	10	10	10	10	10	10	8	10	10	10	10	10	**9**
Disease duration (months)	*r*	-0.134	-0.436	-0.083	-0.149	0.049	-0.402	0.212	0.092	0.263	-0.243	0.097	-0.229	-0.257
	p	0.712	0.208	0.821	0.68	0.893	0.249	0.614	0.801	0.463	0.498	0.789	0.525	0.505
	n	10	10	10	10	10	10	8	10	10	10	10	10	9
King's staging	*r*	**-.756***	0.078	0.576	**.765****	**.708***	-0.628	**.887****	**.921****	**.838****	**.661***	0.261	**.650***	0.52
	p	**0.011**	0.83	0.082	**0.01**	**0.022**	0.052	**0.003**	**<.001**	**0.002**	**0.037**	0.467	**0.042**	0.152
	n	**10**	10	10	**10**	**10**	10	**8**	**10**	**10**	**10**	10	**10**	9
ALSFRS-R total subscore (/48)	*r*	0.621	0.081	-0.365	-0.165	-0.586	0.541	-0.386	-0.337	-0.564	-0.573	0.055	-0.349	-0.469
	p	0.055	0.823	0.3	0.648	0.075	0.106	0.346	0.34	0.089	0.083	0.88	0.324	0.202
	n	10	10	10	10	10	10	8	10	10	10	10	10	9
ALSFRS-R bulbar subscore (/12)	*r*	0.12	0.094	-0.095	-0.374	-0.316	-0.009	-0.457	-0.502	-0.452	**-.869****	0.593	-0.365	-0.009
	p	0.742	0.796	0.793	0.287	0.374	0.98	0.255	0.14	0.189	**0.001**	0.071	0.3	0.981
	n	10	10	10	10	10	10	8	10	10	**10**	10	10	9
ALSFRS-R Fine Motor subscore (/12)	*r*	**.790****	-0.481	-0.196	-0.359	-0.269	0.464	-0.538	-0.36	-0.438	-0.068	-0.364	-0.111	-0.448

		Creatinine (mol/L)	Albumin (g/L)	Prealbumin (g/L)	Cholesterol (mmol/L)	Triglyceride (mmol/L)	HDL cholesterol (mmol/L) †	LDL cholesterol (mmol/L)	Non-HDL cholesterol (mmol/L)	Total HDL-cholesterol (mmol/L)	Ferritin (µg/L)	Transferrin (g/L)	Retinol-binding protein (mg/L)	eGFR †
	p	**0.007**	0.159	0.588	0.308	0.453	0.176	0.169	0.306	0.206	0.852	0.301	0.759	0.227
	n	**10**	10	10	10	10	10	8	10	10	10	10	10	9
ALSFRS-R Gross Motor subscore (/12)	*r*	0.451	0.301	-0.298	0.206	-0.26	0.577	-0.03	0.098	-0.188	-0.128	-0.214	-0.058	-0.361
	p	0.191	0.398	0.402	0.569	0.468	0.081	0.943	0.787	0.604	0.724	0.553	0.873	0.34
	n	10	10	10	10	10	10	8	10	10	10	10	10	9
ALSFRS-R Respiratory subscore (/12)	*r*	0.257	-0.045	-0.344	-0.232	**-.667** *	0.311	0.089	-0.249	-0.328	-0.431	-0.374	-0.35	-0.397
	p	0.473	0.901	0.33	0.519	**0.035**	0.381	0.835	0.489	0.354	0.214	0.287	0.322	0.29
	n	10	10	10	10	**10**	10	8	10	10	10	10	10	9
ΔALSFRS-R	*r*	0.456	0.478	0.116	0.036	-0.19	0.498	-0.374	-0.226	-0.451	0.055	-0.03	0.225	0.256
	p	0.185	0.162	0.75	0.92	0.599	0.143	0.362	0.531	0.191	0.881	0.934	0.532	0.507
	n	10	10	10	10	10	10	8	10	10	10	10	10	9
**Anthropometric**														
Weight (kg)	*r*	0.395	-0.7	0.289	-0.611	0.439	-0.467	-0.464	-0.275	0.216	0.048	-0.143	0.108	0.217
	p	0.333	0.053	0.487	0.108	0.276	0.243	0.354	0.509	0.608	0.911	0.736	0.799	0.641
	n	8	8	8	8	8	8	6	8	8	8	8	8	7
Percentage weight change from premorbid (%)	*r*	**.829** *	0.185	0.029	-0.714	-0.334	-0.058	-0.754	-0.725	-0.486	-0.143	0.086	-0.086	0.335
	p	**0.042**	0.725	0.957	0.111	0.518	0.913	0.084	0.103	0.329	0.787	0.872	0.872	0.581
	n	**6**	6	6	6	6	6	6	6	6	6	6	6	5
BMI (kg/m^2)	*r*	0	**-.883** **	-0.333	-0.193	0.344	0.139	0.058	0.018	0.307	0.311	-0.563	-0.235	-0.477
	p	1	**0.004**	0.42	0.647	0.405	0.744	0.913	0.966	0.459	0.453	0.146	0.575	0.279

		Creatinine (mol/L)	Albumin (g/L)	Prealbumin (g/L)	Cholesterol (mmol/L)	Triglyceride (mmol/L)	HDL cholesterol (mmol/L) †	LDL cholesterol (mmol/L)	Non-HDL cholesterol (mmol/L)	Total HDL-cholesterol (mmol/L)	Ferritin (µg/L)	Transferrin (g/L)	Retinol-binding protein (mg/L)	eGFR †
	n	8	**8**	8	8	8	8	6	8	8	8	8	8	7
Left MUAC (cm)	*r*	0.181	**-.821****	0.212	-0.356	0	-0.206	-0.067	-0.157	-0.015	0.177	-0.312	0.11	-0.037
	p	0.617	**0.004**	0.556	0.313	1	0.569	0.875	0.665	0.966	0.624	0.38	0.761	0.925
	n	10	**10**	10	10	10	10	8	10	10	10	10	10	9
Right MUAC (cm)	*r*	-0.085	**-.864****	0.171	-0.152	0.055	-0.158	0.241	-0.024	-0.012	0.261	-0.345	0.182	-0.219
	p	0.815	**0.001**	0.637	0.675	0.88	0.663	0.565	0.947	0.973	0.467	0.328	0.614	0.571
	n	10	**10**	10	10	10	10	8	10	10	10	10	10	9
Average MUAC (cm)	*r*	0.024	**-.901****	0.171	-0.304	-0.006	-0.219	0.06	-0.128	-0.018	0.212	-0.345	0.097	-0.146
	p	0.947	**<.001**	0.637	0.393	0.987	0.544	0.887	0.724	0.96	0.556	0.328	0.789	0.708
	n	10	**10**	10	10	10	10	8	10	10	10	10	10	9
Left TSF (mm)	*r*	-0.66	-0.205	0.282	0.218	0.521	-0.259	0.523	0.311	0.405	0.259	0.134	0.303	0.342
	p	0.053	0.596	0.463	0.572	0.15	0.5	0.228	0.415	0.279	0.5	0.731	0.429	0.406
	n	9	9	9	9	9	9	7	9	9	9	9	9	8
Right TSF (mm)	*r*	-0.544	-0.613	-0.126	-0.042	0.343	-0.133	0.6	0.226	0.462	0.333	-0.583	0.025	-0.266
	p	0.13	0.079	0.748	0.915	0.366	0.732	0.154	0.559	0.21	0.381	0.099	0.949	0.524
	n	9	9	9	9	9	9	7	9	9	9	9	9	8
Average TSF (mm)	*r*	-0.586	-0.502	0.042	-0.167	0.301	-0.25	0.491	0.042	0.311	0.083	-0.25	0.033	0.051
	p	0.097	0.168	0.915	0.667	0.431	0.516	0.263	0.915	0.415	0.831	0.516	0.932	0.905
	n	9	9	9	9	9	9	7	9	9	9	9	9	8
Left AMA (cm^2)	*r*	0.502	-0.502	-0.025	-0.159	-0.184	0.05	0	-0.042	-0.168	0.017	-0.35	0	-0.381

		Creatinine (mol/L)	Albumin (g/L)	Prealbumin (g/L)	Cholesterol (mmol/L)	Triglyceride (mmol/L)	HDL cholesterol (mmol/L) †	LDL cholesterol (mmol/L)	Non-HDL cholesterol (mmol/L)	Total HDL-cholesterol (mmol/L)	Ferritin (μg/L)	Transferrin (g/L)	Retinol-binding protein (mg/L)	eGFR †
	p	0.168	0.168	0.949	0.683	0.635	0.898	1	0.915	0.666	0.966	0.356	1	0.352
	n	9	9	9	9	9	9	7	9	9	9	9	9	8
Right AMA (cm^2)	*r*	0.033	**-.707***	0.268	-0.017	0.109	-0.3	0.164	0.109	-0.034	0.183	-0.05	0.209	-0.14
	p	0.932	**0.033**	0.486	0.966	0.781	0.433	0.726	0.781	0.932	0.637	0.898	0.589	0.742
	n	9	**9**	9	9	9	9	7	9	9	9	9	9	8
Average AMA (cm^2)	*r*	0.31	**-.707***	0.092	-0.226	-0.1	-0.117	-0.055	-0.109	-0.218	-0.033	-0.2	0.042	-0.342
	p	0.417	**0.033**	0.814	0.559	0.797	0.765	0.908	0.781	0.572	0.932	0.606	0.915	0.406
	n	9	**9**	9	9	9	9	7	9	9	9	9	9	8
Left calf circumference (cm)	*r*	-0.219	-0.466	**.652***	-0.286	0.46	**-.754***	0.048	0.024	0.305	0.188	-0.018	0.456	0.566
	p	0.544	0.175	**0.041**	0.424	0.181	**0.012**	0.91	0.947	0.392	0.603	0.96	0.185	0.112
	n	10	10	**10**	10	10	**10**	8	10	10	10	10	10	9
Right calf circumference (cm)	*r*	0.274	-0.342	0.067	-0.584	0.239	-0.334	-0.422	-0.226	0.238	-0.018	-0.285	-0.091	0.128
	p	0.444	0.334	0.854	0.077	0.506	0.345	0.298	0.531	0.508	0.96	0.425	0.802	0.743
	n	10	10	10	10	10	10	8	10	10	10	10	10	9
Average calf circumference (cm)	*r*	0	-0.441	0.488	-0.468	0.288	**-.657***	-0.096	-0.116	0.262	0.127	-0.176	0.255	0.493
	p	1	0.202	0.153	0.172	0.419	**0.039**	0.82	0.75	0.464	0.726	0.627	0.476	0.178
	n	10	10	10	10	10	**10**	8	10	10	10	10	10	9

Correlation analysis was conducted using Spearman's rank-order correlation analysis for non-normally distributed data. Significance observed at $p < 0.05$. Significant results highlighted in bold. ALSFRS-R: amyotrophic lateral sclerosis functional rating scale - revised; AMA: arm muscle

area; BMI: body mass index; MUAC: Mid-upper arm circumference; TSF: triceps skinfold thickness; ΔALSFRS-R: change in the amyotrophic lateral sclerosis functional rating scale - revised.

Supplementary Table S2. Longitudinal serum analyte concentrations for the group without inflammation.

					P value		
	**M0**	**M3**	**M6**	**M9**	**M0-3**	**M0-6**	**M0-9**
**Creatinine (mol/L) #**	N =10<br>69.50 ± 11.34<br>68.00 (63.50-77.00)	N = 9<br>65.78 ± 9.77<br>65.00 (58.00-71.50)	N = 7<br>73.14 ± 12.76<br>67.00 (64.00-84.00)	N = 6<br>66.67 ± 15.12<br>63.50 (56.50-80.75)	**0.02**	0.69	0.19
**Albumin (g/L)**	N = 10<br>45.90 ± 1.79<br>45.50 (44.75-48.00)	N = 9<br>45.67 ± 2.96<br>46.00 (44.00-48.00)	N = 7<br>46.29 ± 3.15<br>46.00 (43.00-49.00)	N = 6<br>45.33 ± 3.45<br>45.50 (43.00-47.75)	0.98	0.98	0.98
**Prealbumin (g/L)**	N = 10<br>0.27 ± 0.05<br>0.27 (0.23-0.30)	N = 8<br>0.27 ± 0.06<br>0.27 (0.21-0.30)	N = 6<br>0.29 ± 0.04<br>0.31 (0.25-0.32)	N = 5<br>0.28 ± 0.06<br>0.28 (0.23-0.34)	>0.99	0.09	0.80
**Cholesterol (mmol/L)**	N = 10<br>5.22 ± 0.86<br>5.20 (4.45-5.78)	N = 9<br>4.97 ± 0.73<br>5.00 (4.40-5.60)	N = 7<br>5.04 ± 0.85<br>5.00 (4.70-5.50)	N = 6<br>5.15 ± 0.78<br>5.15 (4.60-5.70)	0.74	0.99	>0.99
**Triglyceride (mmol/L)**	N = 10<br>1.49 ± 0.68<br>1.50 (0.95-1.95)	N = 9<br>1.51 ± 0.71<br>1.10 (0.95-2.20)	N = 7<br>1.33 ± 0.77<br>1.10 (0.80-1.60)	N = 6<br>1.78 ± 1.05<br>1.50 (1.05-2.38)	0.92	0.96	0.85
**HDL cholesterol (mmol/L) #**	N = 10<br>1.82 ± 1.02<br>1.47 (1.23-2.03)	N = 9<br>1.46 ± 0.35<br>1.37 (1.22-1.84)	N = 7<br>1.65 ± 0.39<br>1.55 (1.29-2.09)	N = 6<br>1.37 ± 0.24<br>1.29 (1.19-1.54)	0.23	0.81	**0.03**
**LDL cholesterol (mmol/L)**	N = 8<br>2.90 ± 0.86<br>2.85 (2.20-3.30)	N = 7<br>2.59 ± 0.75<br>2.50 (2.00-3.40)	N = 6<br>2.78 ± 0.80<br>2.85 (2.18-3.38)	N = 6<br>2.98 ± 0.68<br>3.10 (2.55-3.48)	0.92	0.83	0.83
**Non-HDL cholesterol (mmol/L) #**	N = 10<br>3.71 ± 1.03<br>3.70 (2.80-4.53)	N = 9<br>3.51 ± 0.83<br>3.80 (2.90-4.10)	N = 7<br>3.41 ± 0.78<br>3.60 (3.30-3.70)	N = 6<br>3.78 ± 0.97<br>3.80 (3.18-4.50)	0.63	>0.99	>0.99
**Total HDL cholesterol ratio (mmol/L)**	N = 10<br>3.71 ± 1.20<br>3.70 (2.58-4.78)	N = 9<br>3.60 ± 0.97<br>3.80 (2.75-4.10)	N = 7<br>3.19 ± 0.71<br>3.40 (2.50-3.80)	N = 6<br>3.92 ± 1.06<br>3.80 (3.33-4.85)	0.99	0.99	0.59
**Ferritin (µg/L)**	N = 10<br>243.20 ± 76.62<br>240.00 (179.75-305.50)	N = 9<br>242.67 ± 87.56<br>272.00 (152.00-320.50)	N = 7<br>241.71 ± 115.79<br>291.00 (123.00-328.00)	N = 6<br>273.17 ± 89.46<br>313.00 (184.50-340.75)	0.81	0.75	0.22

	M0	M3	M6	M9	P value M0-3	P value M0-6	P value M0-9
**Transferrin (g/L)**	N = 10<br>2.27 ± 0.20<br>2.31 (2.07-2.49)	N = 7<br>2.35 ± 0.35<br>2.30 (2.17-2.70)	N = 6<br>2.33 ± 0.43<br>2.30 (2.11-2.53)	N = 6<br>2.34 ± 0.22<br>2.31 (2.12-2.56)	0.89	>0.99	>0.99
**Retinol binding protein (mg/L)**	N = 10<br>50.80 ± 12.25<br>45.00 (40.75-61.75)	N = 8<br>43.15 ± 21.30<br>46.50 (33.00-60.00)	N = 7<br>54.00 ± 10.65<br>53.00 (47.00-63.00)	N = 5<br>54.60 ± 12.40<br>56.00 (42.00-66.50)	0.38	0.13	0.17
**eGFR EPI #**	N = 9<br>86.44 ± 6.64<br>90.00 (84.50-90.00)	N = 9<br>87.00 ± 5.87<br>90.00 (85.00-90.00)	N = 6<br>84.50 ± 10.80<br>90.00 (78.75-90.00)	N = 5<br>86.00 ± 8.94<br>90.00 (90.00-90.00)	>0.99	0.50	>0.99

Data is presented as mean ± one standard deviation and median (IQR). The number of participants with measurements for each biochemical analyte is indicated by ‘n =’. Changes in longitudinal data were analysed for significance using Dunnett’s multiple comparisons test for normally distributed data, or the Wilcoxon matched-pairs signed rank test for non-normally distributed data (#). Significance observed at $p < 0.05$, highlighted in bold. IQR: inter-quartile range; kcal/day: kilocalories per day; M0-9: Months 0-9; SD: standard deviation.

Supplementary Table S3. Longitudinal serum analyte concentrations for the entire cohort.

	M0	M3	M6	M9	P value M0-M3	P value M0-M6	P value M0-M9
**Creatinine (mol/L)**	n = 19<br>66.63 ± 17.54<br>65.00 (53.00-74.00)	n = 17<br>66.59 ± 17.92<br>61.00 (56.00-75.00)	n = 13<br>68.09 ± 18.50<br>64.00 (55.00-78.00)	n = 10<br>59.40 ± 15.81<br>60.00 (45.25-69.75)	n = 15<br>**0.02**	n = 12<br>0.42	n = 10<br>**0.03**
**Albumin (g/L)**	n = 19<br>46.47 ± 2.27<br>46.00 (45.00-48.00)	n = 17<br>46.29 ± 2.52<br>46.00 (45.00-47.50)	n = 13<br>46.31 ± 2.43<br>47.00 (44.50-47.50)	n = 10<br>46.20 ± 3.29<br>46.00 (44.00-47.75)	n = 15<br>0.98	n = 12<br>0.99	n = 10<br>0.96
**Prealbumin (g/L)**	n = 19<br>0.27 ± 0.04<br>0.28 (0.23-0.30)	n = 16<br>0.27 ± 0.05<br>0.27 (0.23-0.39)	n = 12<br>0.29 ± 0.05<br>0.29 (0.25-0.33)	n = 9<br>0.28 ± 0.05<br>0.28 (0.24-0.33)	n = 14<br>0.98	n = 11<br>0.18	n = 9<br>0.61
**Cholesterol (mmol/L)**	n = 19<br>4.97 ± 0.93<br>5.00 (4.40-5.50)	n = 17<br>4.91 ± 1.03<br>4.90 (4.30-5.60)	n = 13<br>4.89 ± 0.84<br>5.00 (4.40-5.35)	n = 10<br>4.98 ± 0.66<br>4.95 (4.40-5.43)	n = 15<br>0.96	n = 12<br>0.96	n = 10<br>>0.99
**Triglyceride (mmol/L)**	n = 19<br>1.49 ± 0.61<br>1.50 (1.00-1.90)	n = 17<br>1.57 ± 0.68<br>1.40 (1.05-2.05)	n = 13<br>1.58 ± 0.70<br>1.40 (1.00-1.95)	n = 10<br>1.71 ± 0.83<br>1.50 (1.18-2.00)	n = 15<br>0.20	n = 12<br>0.24	n = 10<br>0.12
**HDL cholesterol (mmol/L)**	n = 19<br>1.67 ± 0.80<br>1.49 (1.24-1.90)	n = 17<br>1.46 ± 0.44<br>1.37 (1.20-1.83)	n = 13<br>1.52 ± 0.46<br>1.55 (1.23-1.86)	n = 10<br>1.44 ± 0.35<br>1.38 (1.19-1.83)	n = 15<br>0.15	n = 12<br>0.27	n = 10<br>**0.002**
**LDL cholesterol (mmol/L)**	n = 17<br>2.67 ± 0.78<br>2.50 (2.10-3.25)	n = 12<br>2.63 ± 0.83<br>2.50 (2.03-3.30)	n = 12<br>2.63 ± 0.74<br>2.60 (2.10-3.13)	n = 10<br>2.77 ± 0.59<br>2.70 (2.43-3.40)	n = 9<br>0.99	n = 11<br>0.99	n = 9<br>0.91
**Non-HDL cholesterol (mmol/L)**	n = 19<br>3.46 ± 0.90<br>3.40 (2.80-3.90)	n = 17<br>3.45 ± 0.88<br>3.70 (2.65-4.10)	n = 13<br>3.78 ± 0.69<br>3.30 (3.20-3.70)	n = 10<br>3.55 ± 0.80<br>3.55 (3.10-4.08)	n = 15<br>>0.99	n = 12<br>0.97	n = 10<br>0.97
**Total HDL cholesterol ratio (mmol/L)**	n = 19<br>3.51 ± 1.06<br>3.10 (2.70-4.50)	n = 17<br>3.58 ± 1.01<br>3.70 (2.70-4.10)	n = 13<br>3.47 ± 0.95<br>3.40 (2.75-4.10)	n = 10<br>3.70 ± 1.12<br>3.70 (2.70-4.85)	n = 15<br>0.93	n = 12<br>0.99	n = 10<br>0.81

	M0	M3	M6	M9	P value M0-M3	P value M0-M6	P value M0-M9
**Ferritin (µg/L)**	n = 19<br>251.68 ± 114.09<br>226.00 (155.00-337.00)	n = 17<br>240.94 ± 93.79<br>248.00 (161.50-315.00)	n = 13<br>226.62 ± 100.75<br>250.00 (123.00-308.50)	n = 10<br>261.70 ± 102.21<br>277.50 (184.50-340.75)	n = 15<br>0.58	n = 12<br>0.29	n = 10<br>0.82
**Transferrin (g/L)**	n = 19<br>2.39 ± 0.32<br>2.35 (2.09-2.62)	n = 15<br>2.46 ± 0.35<br>2.41 (2.18-2.70)	n = 12<br>2.38 ± 0.37<br>2.34 (2.24-2.67)	n = 10<br>2.37 ± 0.26<br>2.39 (2.12-2.56)	n = 13<br>0.48	n = 11<br>>0.99	n = 10<br>0.98
**Retinol binding protein (mg/L)**	n = 19<br>53.53 ± 12.22<br>55.00 (41.00-65.00)	n = 15<br>51.01 ± 19.48<br>57.00 (38.00-65.00)	n = 13<br>55.62 ± 13.16<br>54.00 (45.50-67.00)	n = 8<br>55.75 ± 9.60<br>57.00 (46.75-63.75)	n = 13<br>0.82	n = 12<br>0.35	n = 8<br>0.44
**eGFR EPI**	n = 18<br>86.06 ± 9.79<br>90.00 (88.25-90.00)	n = 15<br>84.27 ± 10.71<br>90.00 (82.00-90.00)	n = 12<br>84.33 ± 10.89<br>90.00 (84.25-90.00)	n = 8<br>87.38 ± 7.03<br>90.00 (89.25-90.00)	n = 10<br>>0.99	n = 8<br>0.63	n = 6<br>0.75

Data presented as mean ± one standard deviation and median (IQR). The number of participants with measurements for each analyte is indicated by ‘n =’. Longitudinal data was analysed for significance using the Dunnett’s multiple comparisons test for normally distributed data, or the Wilcoxon matched-pairs signed rank test for non-normally distributed data ($^{\#}$). Significance observed at $p < 0.05$, highlighted in bold. IQR: inter-quartile range; kcal/day: kilocalories per day; M0-9: Months 0-9; SD: standard deviation.

Supplementary Table S4. Longitudinal serum analyte concentrations for the group with inflammation.

	M0	M3	M6	M9	P value		
					**M0-M3**	**M0-M6**	**M0-M9**
**Creatinine (mol/L)** #	N = 9<br>63.44 ± 22.93<br>56.00 (50.50-66.0)	N = 6<br>55.33 ± 12.71<br>54.00 (43.75-64.25)	N = 5<br>53.40 ± 10.41<br>53.00 (44.00-63.00)	N = 4<br>48.50 ± 10.21<br>45.50 (40.75-59.25)	0.44	0.63	0.25
**Albumin (g/L)** #	N = 9<br>47.11 ± 2.67<br>46.00 (45.50-48.50)	N = 6<br>46.83 ± 2.14<br>46.00 (45.75-48.00)	N = 5<br>46.20 ± 1.64<br>47.00 (44.50-47.50)	N = 4<br>47.50 ± 3.00<br>46.00 (46.00-50.50)	0.81	0.50	0.75
**Prealbumin (g/L)**	N = 9<br>0.27 ± 0.05<br>0.28 (0.24-0.31)	N = 6<br>0.26 ± 0.04<br>0.27 (0.22-0.29)	N = 5<br>0.27 ± 0.05<br>0.28 (0.22-0.31)	N = 4<br>0.28 ± 0.04<br>0.28 (0.24-0.31)	>0.99	0.82	0.88
**Cholesterol (mmol/L)**	N = 9<br>4.69 ± 0.97<br>4.90 (4.05-5.35)	N = 6<br>4.92 ± 1.37<br>4.70 (4.00-5.93)	N = 5<br>4.50 ± 0.79<br>4.90 (3.70-5.10)	N = 4<br>4.73 ± 0.38<br>4.70 (4.40-5.08)	0.81	>0.99	0.69
**Triglyceride (mmol/L)** #	N = 9<br>1.50 ± 0.56<br>1.50 (1.00-1.85)	N = 6<br>1.77 ± 0.74<br>1.45 (1.35-2.23)	N = 5<br>1.88 ± 0.59<br>1.70 (1.40-2.45)	N = 4<br>1.60 ± 0.48<br>1.45 (1.25-2.10)	0.06	0.13	0.25
**HDL cholesterol (mmol/L)** #	N = 9<br>1.52 ± 0.48<br>1.61 (1.05-1.93)	N = 6<br>1.54 ± 0.63<br>1.73 (0.78-1.97)	N = 5<br>1.39 ± 0.57<br>1.68 (0.77-1.86)	N = 4<br>1.54 ± 0.49<br>1.72 (1.02-1.87)	0.50	0.19	0.13
**LDL cholesterol (mmol/L)**	N = 9<br>2.47 ± 0.68<br>2.40 (2.05-3.00)	N = 4<br>2.98 ± 0.96<br>2.75 (2.20-3.98)	N = 5<br>2.24 ± 0.44<br>2.40 (1.80-2.60)	N = 4<br>2.45 ± 0.17<br>2.50 (2.28-2.58)	0.79	0.93	0.65
**Non-HDL cholesterol (mmol/L)** #	N = 9<br>3.18 ± 0.66<br>3.40 (2.50-3.65)	N = 6<br>3.38 ± 0.86<br>3.35 (2.68-3.98)	N = 5<br>3.10 ± 0.34<br>3.20 (2.85-3.30)	N = 4<br>3.20 ± 0.33<br>3.20 (2.90-3.50)	0.28	>0.99	>0.99
**Total HDL cholesterol ratio (mmol/L)** #	N = 9<br>3.29 ± 0.89<br>3.10 (2.70-3.60)	N = 6<br>3.48 ± 1.10<br>3.00 (2.75-4.38)	N = 5<br>3.60 ± 1.15<br>2.90 (2.75-4.80)	N = 4<br>3.38 ± 1.28<br>2.75 (2.70-4.68)	0.16	0.31	>0.99
**Ferritin (µg/L)**	N = 9<br>261.11 ± 149.97<br>226.00 (151.50-412.50)	N = 6<br>235.17 ± 124.67<br>222.00 (127.50-351.25)	N = 5<br>200.80 ± 94.73<br>206.00 (107.50-291.50)	N = 4<br>244.50 ± 131.40<br>239.00 (121.50-373.00)	0.76	0.99	0.28

	M0	M3	M6	M9	P value M0-M3	M0-M6	M0-M9
**Transferrin (g/L)**	N = 9<br>2.52 ± 0.39<br>2.62 (2.13-2.85)	N = 6<br>2.48 ± 0.30<br>2.54 (2.16-2.73)	N = 5<br>2.46 ± 0.37<br>2.43 (2.14-2.80)	N = 4<br>2.42 ± 0.34<br>2.45 (2.10-2.72)	>0.99	0.56	0.61
**Retinol binding protein (mg/L)**	N = 9<br>56.56 ± 12.16<br>58.00 (46.00-65.00)	N = 5<br>54.20 ± 10.62<br>57.00 (4.00-63.00)	N = 5<br>53.60 ± 15.03<br>59.00 (38.50-66.00)	N = 3<br>57.67 ± 2.52<br>58.00 (55.00-60.00)	0.77	0.94	0.39
**eGFR EPI** #	N = 9<br>85.67 ± 12.63<br>90.00 (89.50-90.00)	N = 4<br>90.00 ± 0.00<br>90.00 (90.00-90.00)	N = 5<br>89.00 ± 2.24<br>90.00 (87.50-90.00)	N = 3<br>89.67 ± 0.58<br>90.00 (89.00-90.00)	>0.99	>0.99	!

Data is presented as mean ± one standard deviation and median (IQR). The number of participants with measurements for each biochemical analyte is indicated by ‘n =’. Changes in longitudinal data were analysed for significance using the Dunnett’s multiple comparisons test for normally distributed data, or the Wilcoxon matched-pairs signed rank test for non-normally distributed data (#). Significance observed at $p < 0.05$, highlighted in bold. IQR: inter-quartile range; kcal/day: kilocalories per day; M0-9: Months 0-9; SD: standard deviation; !: data has zero difference.
